# Comparing Efficacy Between Regimens in the Initial Treatment of Autoimmune Hepatitis

**DOI:** 10.4021/jocmr1486w

**Published:** 2013-06-21

**Authors:** Chijioke Enweluzo, Fahad Aziz, Amit Mori

**Affiliations:** a Section on Hospital Medicine, Department of Internal Medicine, Wake Forest School of Medicine, Medical Center Boulevard, Winston Salem, NC 27101, USA

**Keywords:** Autoimmune hepatitis, Efficacy, Treatment, Prednisone, Azathioprine, Complete resolution, Incomplete resolution

## Abstract

**Background:**

Autoimmune hepatitis is a chronic syndrome characterized by auto immunologic features generally including the presence of circulating auto antibodies and high serum globulin concentrations. The American Association for the Study of Liver Diseases (AASLD) recommends initial treatment or induction therapy for autoimmune hepatitis to involve a glucocorticoid alone or a combination of a glucocorticoid and an immunosuppressant. The objective of this study is to review and compare the efficacy of the treatment regimens described above among patients diagnosed with and treated for autoimmune hepatitis over the past 10 years in our center which is a major university based hospital.

**Methods:**

We retrospectively identified patients above the age of 18 years diagnosed with autoimmune hepatitis in our center between February, 2003 and February, 2013 using the ICD-9 code 571.42. The primary outcome of our study was efficacy of the treatment regimen. We defined efficacy by considering 3 scenarios: Complete Resolution, Incomplete Resolution and Treatment Failure.

**Results:**

We found differences among 3 treatment groups: patients who received Prednisone and immunosuppressant from the beginning of their treatment course, patients who had an immunosuppressant introduced after about 4 weeks on Prednisone and patients who were placed on Prednisone alone.

**Conclusion:**

From our study, better efficacy was achieved in the induction phase using a combination of Prednisone and Azathioprine from the beginning of the treatment course.

## Introduction

Autoimmune hepatitis is a chronic syndrome characterized by auto immunologic features generally including the presence of circulating auto antibodies and high serum globulin concentrations [1]. It is a disease that is protean in terms of presentation, clinical, serological and histological features with a fairly rapid progression to cirrhosis and high mortality rate if left untreated. Common presenting symptoms are non specific and include fatigue, nausea abdominal pain, aching joints, and itching. Other common symptoms include jaundice, hepatosplenomegaly and spider angiomas on the skin. Other symptoms may include dark urine, loss of appetite, pale stools and absence of menstruation. In up to 20% of cases, autoimmune hepatitis may present with symptoms like an acute hepatitis [2]. It is generally thought to be a steroid-responsive condition where early diagnosis and early institution of appropriate therapy result in favorable outcomes. The American Association for the Study of Liver Diseases (AASLD) recommends initial treatment or induction therapy for autoimmune hepatitis to involve a glucocorticoid alone (monotherapy) or a combination of a glucocorticoid and an immunosuppressant such as Azathioprine, CellCept or 6-Mercaptopurine (dual therapy) [3]. The dual therapy allows use of lower doses of glucocorticoid thereby mitigating steroid side effects. Immunosuppresants are also added when there is incomplete or no response to monotherapy. Lammers MM et al, reported similar efficacy between both regimens for induction therapy even though their study also acknowledges the paucity of data and research in this regard [4]. Up to 90 percent of patients with moderate to severe autoimmune hepatitis will respond to treatment, with a decrease in serum transaminases along with symptom improvement within two weeks. In the majority of these patients, the serum transaminases will fall into the normal range, generally after 12 or more months of treatment. However, clinical, laboratory, and histologic parameters improve but fail to normalize (incomplete response) in 13% of patients, and they worsen in 10% (treatment failure) [4, 5]. The objective of this study is to review and compare the efficacy of the treatment regimens described above among patients diagnosed with and treated for autoimmune hepatitis over the past 10 years in our center which is a major university based hospital.

## Methods

We retrospectively identified patients above the age of 18 years diagnosed with autoimmune hepatitis in our center between February, 2003 and February, 2013 using the ICD-9 code 571.42. A chart review was carried out and data regarding patients’ demographics in terms of age, gender and year of diagnosis, presence of transaminitis, auto antibodies and diagnosis by liver biopsy was collected. Additionally, the type of initial therapy instituted, presence of complete resolution, incomplete response to therapy or treatment failure and eventual development of cirrhosis were also obtained. Patients diagnosed with viral hepatitis, overlap syndrome, alcoholic liver disease, fulminant hepatitis from any cause as well as patients with granulomatous hepatitis secondary to any cause were excluded from the study. Patients diagnosed with autoimmune hepatitis and receiving treatment of either kind for less than 2 years were also excluded from the study [6]. We also excluded patients who were diagnosed with autoimmune hepatitis and did not receive treatment of any kind.

The primary outcome of our study was efficacy of the treatment regimen. In determining efficacy, we considered 3 scenarios [2, 7, 8]: 1), complete Resolution which was defined as resolution of symptoms and normalization of transaminitis as well as serum bilirubin and gamma globulin levels; 2), incomplete response to therapy which was defined as some or no improvement in clinical, laboratory, and histologic features despite compliance with therapy for two to three years; 3), treatment failure which was characterized by sustained biochemical and histologic activity, leading to the development or worsening of cirrhosis with eventual complications and death or the need for orthotopic liver transplantation.

The study was approved by the Wake Forest University Baptist Medical Center Institutional Review Board.

## Results

### Patient demographics

**Figure 1:**
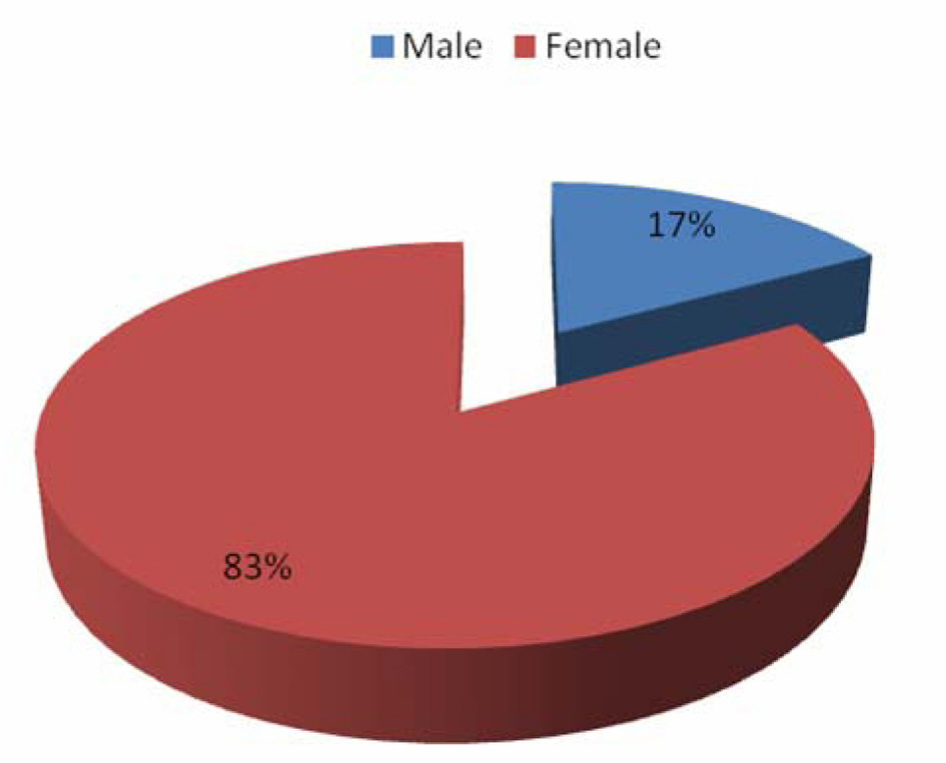
Patient distribution by gender.

**Figure 2:**
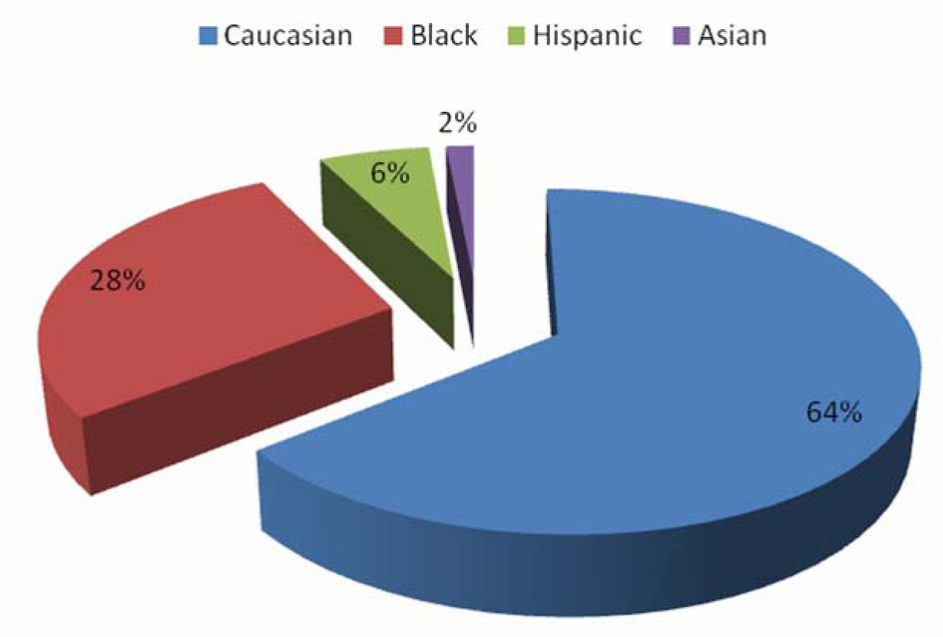
Patient distribution by race.

A total of 133 patients who were diagnosed and treated for autoimmune hepatitis within the specified time period were identified. Our study population was mostly female with a female-to-male ratio of 6:1 (Fig. 1). There was a wide age range (18 - 78 years) with a mean age of 52 years, 64% of our patients were Caucasian (n = 85), 28% were Black (n = 37), 6% were Hispanic (n = 9) while 2% (n = 2) were Asian (Fig. 2).

### Diagnosis

An incidental finding of transaminitis on routine work up or during investigation for other conditions was the first indication of liver disease in majority of patients leading to further analysis. This was seen in 98% of patients (n = 130). The remaining 2% (n = 3) presented with jaundice and abdominal pain, 93% (n = 124) of our patients demonstrated elevated IgG concentration prior to therapy, 86% (n = 114) also tested positive for immunological markers like ANA and ASMA. All patients tested negative for any viral hepatitis markers. All study patients had a liver biopsy prior to onset of treatment with resultant histological patterns confirming diagnosis of autoimmune hepatitis and excluding cirrhosis. In terms of Immunosuppresants, we found Azathioprine to be the drug of choice here being used in 96% (n = 128) of our patients. Alternative regimens were only used in patients with documented allergies or contra indications to Azathioprine.

### Treatment and outcomes

**Figure 3:**
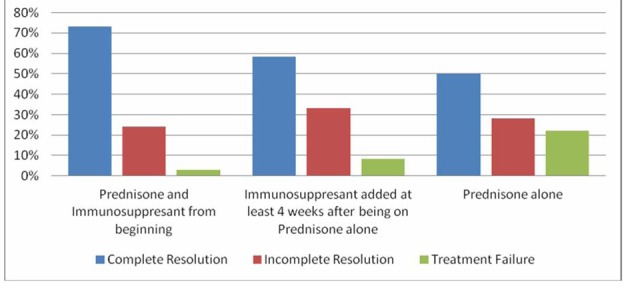
Comparison between treatment groups.

47.4% of the 133 patients in the study (n = 63) received prednisone and an immunosuppressant from the beginning of treatment. Of these 63 patients, 73% (n = 46) experienced complete resolution leading to a reduction and in some cases, discontinuation of Prednisone to minimize steroid side effects, 24% (n = 15) patients achieved incomplete resolution while 3% (n = 2) patients had treatment failure (Fig. 3). The decision to treat with both Prednisone and an immunosuppressant from the beginning was mainly due to physician preference, 36% of the 133 patients in the study (n = 48) were initially started on prednisone alone with varying degrees of response. An immunosuppressant was subsequently added after at least 4 weeks of Prednisone monotherapy, 58.3% of these 48 patients (n = 28) achieved complete resolution following addition of the immunosuppressant while 33.3% (n = 16) had incomplete resolution and 8.4% of these patients (n = 4) developed cirrhosis indicating treatment failure (Fig. 3), 16.4% of the total number of patients in the study (n = 22) received only prednisone throughout the course of their treatment. These patients were never started on any immunosuppressant, 50% of these patients (n = 11) achieved complete resolution and successfully had their steroid doses deescalated, 28% of these patients (n = 6) had incomplete resolution while 22% (n = 5) experienced treatment failure (Fig. 3). We also found 6 patients who did not receive any treatment for various reasons (medical co morbidities or non compliance) and all of them developed liver cirrhosis. Across all categories, 8.3% of the entire study population (n = 11) eventually developed cirrhosis at the time of this study.

## Discussion

Autoimmune hepatitis remains a disease with an unpredictable and in some instances, unresolving course. As noted before, the AASLD recommends induction/initial therapy with Prednisone alone or the combination of Azathioprine to Prednisone. Azathioprine monotherapy is only recommended for maintenance therapy after initial therapy. The major advantage of the dual therapy is to mitigate the side effects of steroids especially in the long term as this is a disease that in many cases needs some form of maintenance treatment for continued remission. On the other hand, Azathioprine and its drug metabolite 6-Mercaptopurine are not without their side effects. They should be used with great caution in patients with pre existing cytopenias, malignancy or thiopirine methyltransferase (TPMT) deficiency. Such patients are typically considered for monotherapy with Prednisone.

The AASLD recommends that treatment be instituted in the following situations [3, 9]: 1), serum alanine aminotransferase (ALT) or serum aspartate aminotransferase (AST) level greater than 10-fold the upper limit of normal; 2), serum ALT, AST, or gamma globulin level greater than twice the upper limit of normal if any of the following are present: (1), symptomatic patients; (2), an elevated conjugated bilirubin or, in the case of an ALT or AST that is twice the upper limit of normal, an elevated gamma globulin level, even if less than twice the upper limit of normal; (3), interface hepatitis on biopsy; (4), histologic features of bridging necrosis or multiacinar necrosis; (5), cirrhosis with any degree of inflammation on biopsy; (6), children with autoimmune hepatitis.

The risks of drug treatment and the possibility of deleterious side effects needs to be cautiously weighed against the benefits of the same in asymptomatic patients with normal serum aminotransferase/gamma globulin levels and evidence of minimal cirrhotic progression despite strong suspicion of autoimmune hepatitis. Nonetheless, such patients need to be placed under close surveillance and treatment started immediately following the advent of abnormal liver enzymes or gamma globulin levels. Close surveillance should also include adequate diet and exercise to prevent obesity and possible concomitant non alcoholic fatty liver disease, proper hepatitis A and B vaccination to prevent concomitant or super infection with viral hepatitis, abstinence from alcohol to prevent alcoholic liver disease and a high importance placed on safe sexual practices. Patients presenting with acute/Fulminant hepatitis secondary to autoimmune hepatitis need to be referred to a liver transplant center for further evaluation and management. The above retrospective study only seeks to assess and compare the efficacy of recommended regimens of the induction phase in the treatment of Autoimmune Hepatitis [10, 11]. Obviously, the goal in treating these patients is to achieve long lasting remission but this is easier said than done. As mentioned before, this is a disease that is as unpredictable as it is severe if not carefully attended to. Our study results show that better efficacy was achieved in the induction phase using a combination of Prednisone and Azathioprine from the beginning of the treatment course. The relatively low number of patients and the fact that the study was performed in a single center are probable limitations. However, we are of the opinion that our results could serve as foundation for further research which can lead to better defined guidelines in the treatment of Autoimmune Hepatitis.
